# The polyfactorial model of autism and the question of causality

**DOI:** 10.3389/fpsyt.2023.1117807

**Published:** 2023-06-27

**Authors:** Bernard Golse

**Affiliations:** Université Paris Cité, Paris, France

**Keywords:** autism, Neuroscience, Psychoanalysis, epistemology, Model, epigenetic

## Abstract

After recalling the different pediatric, psychopathological and child psychiatric models of mental disorders in children and adolescents, the author presents in detail the so-called polyfactorial model, which includes primary, secondary, and mixed factors. This model is the epistemological heir of the Freudian concept of “complementary series.” The example of autism is then explored as a paradigm of the usefulness of this polyfactorial model. Finally, we reflect on the notion of causality, from Aristotelian causality to epigenetic causality, which could 1 day re-legitimize psychoanalysis and the impact of the relationship on genome expression.

## Introduction

The question of epistemological models is important in child psychiatry, on the one hand to think about the question of causality and on the other hand to guide the choice of therapeutic strategies.

Growth and psychological maturation correspond to eminently complex processes and the same is true for developmental disorders.

It is an oversimplification to try to make people believe otherwise, and therefore the management of mental disorders in children and adolescents must carefully consider its reference model(s) (1).

In this work, autism will be taken as the paradigm for reflection, but many of the reflections presented in this respect can, in our opinion, be extended to other child psychiatric disorders.

## The pediatric, psychopathological and child psychiatric models

The question is not whether one model is more valid than another, but to emphasize that each discipline, depending on its practice and objectives, refers to models that are specific to it and therefore useful.

Pediatrics refers to a medical model—like all somatic disciplines—which is rather monofactorial (a single cause is supposed to account for the pathological situation), deductive (based on univocal cause-and-effect relationships) and referring to a linear type of temporality (organized according to the arrow of time in the usual sense of the term).

Psychoanalysis (and psychodynamic psychopathology in general) refers to a different model, of a polyfactorial nature (as S. Freud had proposed it in 1915/17 with his concept of “complementary series”) (2), inferential (proceeding by associations of thoughts and not by deduction), and based on a circular temporality integrating the so-called after effects, according to which the past partly accounts for the present, but the present also allowing, permanently, to retell, rewrite and re-construct the past history.

It should be noted that the deductive somatic model aims for rapid efficiency, whereas the inferential psychopathological model cannot claim to master the tempo of understanding, elaboration and decision.

In any case, the child psychiatric model seeks its place and its identity in relation to these two models.

Depending on the country and the time, the psychiatric model is more or less close to one of the two previous models.

Today, in Anglo-Saxon countries, the child psychiatric model is very close to the medical model (3), whereas in France, it remains, in a way, at equal distance from the medical model and the psychopathological model, still quite strongly impregnated by the psychoanalytical references which presided over the birth of child psychiatry in our country.

It is therefore clear that pediatricians on the one hand, and child psychiatrists, psychologists and child psychoanalysts on the other, do not refer to the same model.

This does not mean that part of the conceptual path cannot be travelled jointly, even if fundamental differences exist. But it does underline the fact that this common path requires mutual esteem and respect between the representatives of the somatic and psychic disciplines.

The difference in tempo often acts as a seed of possible dissension, and in this respect, it is important to say something about the concept of “negative capacity” that Bion (4) developed from the work of the romantic poet J. Keats (5).

This is the clinician’s ability to tolerate ignorance for a certain period of time, not to want to understand everything immediately, to allow himself to be impregnated by the clinical situation, to allow himself to be deeply affected at the level of his emotions, and finally to know how to give time to time so that elaborations, interpretations and conclusions do not have the value of theorizing and defensive force.

This aptitude is necessary to the caretakers of the psyche whereas it is not expected nor even sometimes necessary for caretakers of the body: hence, sometimes, a certain number of possible misunderstandings and specially within the framework of what is called liaison child psychiatry.

## The polyfactorial model

The polyfactorial model probably applies to a number of somatic pathologies (6, 7), but in the field of child and adolescent mental disorders, it seems to us that the most plausible model of the etiology of autistic pathology at present is a polyfactorial model, which is the only one capable of articulating physical causality, interactive causality and epigenetic causality, thereby imposing on us an integrated multidimensional management of this pathology.

In this perspective, autistic functioning would then be a sort of “common final pathway” of a whole series of etiopathogenic configurations within which endogenous and exogenous factors would always be present, but in a variable proportion according to each child.

What is important to specify is the double level of the polyfactorial dimension that can be invoked both for the primary factors of vulnerability, which are always multiple (exogenous and endogenous), and for the secondary factors of fixation and maintenance, among which the deferred action is essential, because the meanings that the first interactive dysfunctions of their child may have for such and such parents can then function autonomously as secondary causal factors, as we shall return to.

This being said, primary factors are only risk factors, whereas secondary factors are factors that fix a psychopathology that is sometimes partially reversible.

It is likely that the years to come will see the emergence and development of the concept of “epigenetic causality” (8) which will finally make it possible to articulate, in a dialectical and singular manner for each patient, the role of internal determinants (the personal part of each person with his or her genetic, neurobiological, cognitive equipment and so on …) and external determinants (the part of the environment in all its components, ecological, dietary, social, cultural, family and so on…) as to the origin of his or her difficulties or developmental disorders.

### Primary and secondary factors

^*^The Freudian concept of “complementary series” (Freud, 1915/1917) mentioned above represents, in a way, the epistemological ancestor of our current polyfactorial model.

This concept constituted, at the time, a real epistemological revolution insofar as it was a profound break with the prevailing medical vision, which was then closely linked to the perspectives developed by Claude Bernard (9).

Freud proposed a sort of power grab by making the hypothesis that the constitution of any neurotic organization could only be conceived as the result of the joint influence of endogenous and exogenous factors.

Among the endogenous factors, he had in mind the fixation points that he had put forward as part of his scheme of psycho-affective development: an oral fixation point predisposed, according to him, to the organization of a hysterical neurosis, an anal fixation point to the organization of an obsessive neurosis, and a phallic or urethro-phallic fixation point to the organization of a phobic or hysterical neurosis.

But, he specified, these fixation points could not be considered as causes in the linear sense of the term, because they were, according to him, only predisposing factors, not determining ones.

In this model, exogenous factors had to precipitate and decompensate things, and among these exogenous factors, he insisted, in a central way, on the question of sexual frustration.

He added that in this model, there was a sort of ‘sliding scale’ between endogenous and exogenous factors, the more important the one being, the less important the other needing to be, but the presence of both being indispensable in each subject to account for his or her psychopathology, according to a pathogenic equation that was therefore strictly specific and individual.

This conception of psychopathological etiology must undoubtedly be clarified; it must however be emphasized that it was extremely innovative at the time, and that our current polyfactorial model derives quite directly from it, even if it has since become much more complex.

* Today, in fact, we consider that any psychopathological situation is the result of the interplay of primary and secondary factors, and that these two lines of factors are each fundamentally polyfactorial in nature. Both primary and secondary factors can, moreover, be of an endogenous nature (personal part of the child) or of an exogenous nature (environment).

Primary factors are only predisposing factors (or vulnerability factors): they are not sufficient, but they are necessary, they do not create psychopathology, but they increase the risk of it.

Secondary factors are decompensating (triggering) factors for psychopathology in subjects who have primary factors that make them vulnerable.

### A paradigmatic example: childhood autism?

By way of illustration, we will now take the example of childhood autism and pervasive developmental disorders, while making it clear that in our opinion, these considerations on autism and autism spectrum disorders have conceptual implications that extend well beyond these various pathologies.

(1) Examples of primary (vulnerability) factors

*– Genetic factors*

These are undeniable, but they are now to be understood from the point of view of genetics of vulnerability, and not causal genetics in the classical sense of the term (10). It is known, for example, that Fragile X syndrome is a predisposing factor for childhood autism, since although in the population of autistic children there is a clear excess of children carrying this anomaly (about 7%) compared to the frequency observed in the general population, conversely not all Fragile X children are autistic.

Furthermore, Kanner’s (11) genetic model of autism as a specified pervasive developmental disorder is currently oriented towards a constellation of alleles involved in so-called “candidate” genes, spread over all the chromosomes and whose joint state would underlie the autistic predisposition. Only this model makes it possible to account for the fact that around an autistic proposer, nearly 1% of other cases are found among first-degree relatives, whereas from the second degree of kinship, the frequency falls back to that observed in the general population (10).

No Mendelian model is capable of explaining this phenomenon, and we are therefore in the perspective of a susceptibility genetics referring to the question of the heritability of complex traits and to epistatic interaction processes (12), which now invite us to classify genetic factors among the so-called primary factors of the polyfactorial model.

Even the recent discoveries (13) on neuroligins and neurexins, which are said to occur in less than 1% of autism cases, should probably be interpreted only in terms of vulnerability.

– *Neurological factors*

The same reasoning as for Fragile X syndrome can be applied to Bourneville Tuberous Sclerosis (BTS): among autistic children, there is a clear increase in the frequency of this condition compared to the general population (A. (14)), but the fact that 25–60% of children affected by this particular encephalopathy are also autistic makes BTS a primary—and only primary - predisposing factor, *via* mutation of the TSC1 (Hamartine) and TSC2 (Tuberine) genes.

The complex problem of epilepsies associated with approximately 30% of autism cases and appearing at some point in the course of the disease must also be mentioned in this section (15). It is still difficult, at the present time, to really specify the intimate mechanisms of this association, which probably differ according to each type of epilepsy, but as far as West syndrome is concerned, it seems that it can be considered as a primary risk factor: it is known that 20–25% of children with West syndrome become autistic in the first 2 years of life, without it being possible to predict in advance which ones, and on the basis of a profound interference with early interactions by certain motor stereotypies of the upper limbs, as Ouss et al. (16) have been able to begin to show in the framework of our research program “PILE” (International Program on Child Language).

As for the anomalies revealed by functional MRI of the superior temporal lobes in children with autism (17), if in some cases they are not only the consequence of autistic-like functioning, they can perhaps be understood as reflecting an important etiopathogenic link, but here we are no longer quite in the framework of primary factors in the strict sense.

*– Sensory factors*

Unlike blindness, deafness appears to be an undeniable risk factor for infantile autism, to the extent that it was thought, at one time, that it was important to identify a specific syndrome associating autism and deafness. Today, while auditory and/or visual cortical hypersensitivity has been demonstrated in the pathogenesis of autism (18), it is thought that deafness plays a primary, non-specific role *via* the partial relational isolation it causes in a large number of cases.

– Infectious factors

Congenital rubella was once shown to occur more frequently in the histories of children with autism than in the general population. The anti-rubella vaccination has somewhat relegated this question to the background, but it remains that the neurological and sensory disorders linked to this embryo-fetopathy could undoubtedly function as primary risk factors.

– Environmental factors, finally

The whole question of interactive dysfunctions and maternal depressions is at stake here. This question must be treated with caution. It is important to consider that these environmental factors can only be considered as primary risk factors within a truly polyfactorial model, i.e., considering that they probably need to be associated with other primary factors (genetic, in particular) to have a real impact. With this proviso, which is essential, we can then imagine that they can play a role as risk factors to be integrated into the “autising process” proposed by Hochmann (19).

This list is by no means exhaustive, and developments in child psychiatric research will very probably lead to new discoveries in this field in the years to come, perhaps with the identification of primary environmental factors in the broad sense (dietary or ecological, for example).

(2) Examples of secondary (triggering) factors

As child psychiatry is still relatively young, these secondary factors are far from being fully identified.

As we have just said with regard to primary factors, the future will certainly allow us to specify, in a wide variety of fields, secondary factors for triggering or decompensating autism in vulnerable children (environmental, nutritional, socio-familial, cultural, ecological, anthropological factors and so on…)

It is now thought that certain perinatal factors, and in particular maternal stress, could play a role in triggering autistic organization in certain babies who are vulnerable in terms of their primary factors.

Certain modifications of the maternal lifestyle as well as perinatal maternal stress as well as sleep disorders and maternal melatonin metabolism could also be invoked (20, 21).

To which we can also add that abnormalities in the general movements of the fetus and the baby during the first months of life (extension reflex and “fidgety” movements in particular) could testify to a neurodevelopmental fragility and interfere with the system of early interactions (A. Beaulieu) while also offering the conditions for an early prevention of the emergence of an autistic organization.

For the time being, the secondary factors that we know best, and on which we can act the most, are the relational factors, i.e., the child’s encounter with the psyche of others, within the framework of the “autising process” mentioned above.

It is not at all a question of reopening the debate on the guilt of families in the genesis of infantile autism, a debate which we know the ravages to which it has given rise (22, 23).

It is clear that parents are in no way responsible or guilty for their child’s autism, which can now only be understood from a polyfactorial perspective.

However, within the framework of a polyfactorial model such as the one presented here, it must be possible to question the status of possible early interactive anomalies as primary or secondary factors, and possibly take them into account as such, either from a prevention (secondary) perspective, or from a care perspective.

(3) Thus, what is fascinating in child psychiatry is that certain factors can play as primary or secondary factors depending on the case, and that it is the role of psychopathological analysis to be able to specify things as finely as possible.

Let us take the case of quantitative or qualitative deficiencies, which have been the subject of particular attention for a number of years.

In some cases, deficiency factors act as primary factors of fragility, and we know, for example, that some children presenting an anaclitic depression, in the sense of Spitz (24), can perfectly well become autistic if the deficiency situation persists and the experience of the orphanages in Romania has again recently provided sad confirmation of this (25–27).

On the other hand, it seems, according to F. Tustin (28), that autistic pathologies are, moreover, quite often the result of the encounter of vulnerable children with an environment that is deficient or insufficiently available, at the interactive level, for this or that reason.

A case-by-case analysis is therefore essential.

All the more so as the frequency of cases of autism seems to be increasing, currently within families in great precariousness (29) and that, among these, the number of migrant families is undoubtedly significant.

As a result, we should not allow ourselves to think, as some authors have hastily done, that migrant families bring with them autism genes; we can see the unjustifiable shortcut that this reasoning makes, with obvious political implications.

The reality is more complex than that and, without doubt, we should rather take into consideration the fact that socio-familial precariousness may well, as a secondary factor, decompensate children who are vulnerable in terms of their primary factors (and in particular genetic factors) within the framework of an epigenetic causality; these children would probably never have become autistic without the encounter with this particular and painful sociological reality.

Taking into account, on the one hand, the intertwining of primary and secondary factors and, on the other hand, the fact that the same factor may intervene, depending on the case, as a primary or secondary factor, seems to us at present the only way to allow room for freedom and a place to the effects of encounters in psychopathological etiopathogeny, which would otherwise risk being reduced to a linear and reductive schema of a strictly endogenous and neurodevelopmental type.

## From Aristotelian causality To epigenetic causality

The central epistemological problem posed by this reductionist view, which is belied by the clinic, paradoxically seems to be often overlooked.

### The different types of causality

Without detailing here the four types of causes that Aristotle (384-322 BC) defines in his Nicomachean Ethics (30) (the material cause, the formal cause, the driving cause and the final cause), we know that from the point of view of a dynamic psychology or psychopathology, it would undoubtedly be interesting to distinguish between the causes that drive (in reference to the drive system) and the causes that attract (in reference to the goal representations).

The drive itself includes its more or less specific goal which pushes the subject to action and the goal-representations (in the Freudian sense of the term) have an effect of attraction which mobilizes the action procedures of this same subject.

But, once again, the essential thing is undoubtedly to resolutely consider a polyfactorial model, both for the approach of development and of developmental disorders.

Within the framework of this polyfactorial model, the notion of interactive causality emerges, involving dialectical effects between the personal part of the subject (i.e., his or her genetic, neurobiological, somatic equipment and so on..) and the role of the environment in all its components (ecological, biological, nutritional, social, family, cultural and so on…). The relational component (i.e., the subject’s encounter with the psychic work of others) has a particularly important function within the environment, and is essential for the disciplines devoted to the functioning of the psyche.

Taking this relational component into account within a polyfactorial model does not mean a return to a purely external causality if one clearly maintains the framework of a polyfactorial model.

In this perspective, there would probably be no pure psychological causality insofar as the impact of the environment depends fundamentally on the personal part of the subject.

Even in the case of traumatic pathology, the study of Post Traumatic Stress Disorders (PTSD) must today consider the subject’s ‘temperament’ for neuroscientists (which corresponds to what psychoanalysts call history and deferred action) in order to understand the different effects of the same trauma on different subjects.

The reign of an exclusive psychogenesis thus appears today to be definitively outdated.

In fact, we would like to insist here on the notion of epigenetic causality, which represents one of the dimensions–probably the most recently discovered—of this interactive causality, a dimension linked to the concept of epigenesis.

### Epigenetic causality In general and In relation To autistic organizations

Psychological development and the disorders of this development are played out at the exact intersection of a certain number of endogenous factors and a certain number of exogenous factors.

There is no room for any kind of division and psychopathology—whether psychoanalytical, attachmentist, systemic, cognitive or developmental—must imperatively integrate this fundamental and founding dialectic between internal and external determinants.

On this basis, many developmental disorders can probably be conceptualized as the result of a mixed epigenetic causality.

What does this term mean?

Usually, when we talk about epigenetic causality, we refer to the influence of the external environment on the expression of the genome.

However, the problem of autistic ontogenesis perhaps also invites us to consider the influence of experiences, feelings and bodily experiences themselves on the expression of the genetic determinants of this epigenesis.

In other words, if it is plausible to imagine that the nature of the child’s early interactions with the adults who take care of them (parents and/or professionals) can have an impact on the expression and regulation of the genetic part that underlies the setting up of an autistic type of organization (we could speak here of external epigenetics), however it is not impossible to think that the bodily and sensory experiences linked to autistic functioning itself may also have an impact on the expression and regulation of the parts of the genome that govern the genetic level of autistic susceptibility (here we could talk about internal epigenetics).

It is the intertwining of these two facets of epigenetics that leads us to propose the term mixed epigenetic causality.

### The causality of autistic pathologies or when The consequences of first causes become second causes

However, as psychoanalyst as one may be, we can affirm today that there is no pure psychogenesis of autism.

Not just anyone becomes autistic, as the study of extreme situations such as the one studied in Romania during the opening of nurseries and orphanages at the end of Ceausescu’s political era has clearly shown.

All the children discovered there were certainly severely deficient in the sense of R. Spitz’s hospitalism (24), but only 30% among them were truly autistic (even if there are areas of symptomatic overlap between deficiency pathology and autistic pathology).

The autistic causality is most likely, as we have seen, polyfactorial, interactive and epigenetic.

This being so, things are even more complex insofar as in the framework of the “autising process” described by Hochmann (19), the consequences of the very first dysfunctions (whether they occur in the baby or in the adult) can then assume the status of second causes and thus turn on a sort of interactive spiral that is dangerously pathogenic and destined to rapidly self-aggravate.

### Can epigenetic causality re-legitimize psychoanalysis?

Lebovici often said (unpublished oral communication) that psychoanalysis had nothing to fear from the current spectacular advances in neuroscience, and that it was even looking forward to them, as they would certainly give us new doors of entry into a polyfactorial model which it is important for us to stand firm, in the field of psychopathology.

It is in this perspective that the following lines are written.

Whatever the future of this hypothesis of a mixed epigenetic causality, which is of course only valid as a conceptual proposal at the present time, we wish to emphasize that psychopathology today can no longer ignore the body, this body which is in essence at the interface of the relationship with the external environment and our internal perceptions forming the basis of our sensoriality, our sensuality and hence our sexuality.

The future of psychoanalysis in a sense is at stake here, because in view of current neurobiological positions, the requalification of speech will probably only be possible through the demonstration (still to come) of its epigenetic effects, which are already plausible.

## Conclusion

Epistemology in psychoanalysis and psychiatry is still insufficiently developed (31); it is nevertheless essential for us to specify the models to which we refer implicitly and/or explicitly, to think about the question of causality and to choose our therapeutic strategies.

We have taken the example of autism, which is known to be a controversial subject, but this epistemological reflection concerns all mental disorders in children and adolescents and can only be effectively conducted in a resolutely transdisciplinary spirit.

## Data availability statement

The original contributions presented in the study are included in the article/Supplementary material, further inquiries can be directed to the corresponding author.

## Author contributions

The author confirms being the sole contributor of this work and has approved it for publication.

## Conflict of interest

The author declares that the research was conducted in the absence of any commercial or financial relationships that could be construed as a potential conflict of interest.

## Publisher’s note

All claims expressed in this article are solely those of the authors and do not necessarily represent those of their affiliated organizations, or those of the publisher, the editors and the reviewers. Any product that may be evaluated in this article, or claim that may be made by its manufacturer, is not guaranteed or endorsed by the publisher.

## References

[1] Ouss-Ryngaert L Golse B. Linking neuroscience and psychoanalysis from a developmental perspective: why and how? J Physiol. (2010) 104:303–8. doi: 10.1016/j.jphysparis.2010.09.005 20888906

[2] Freud S. Point de vue du développement et de la régression–Étiologie, 319-336 et Les modes de formation de symptômes In: Freud S, editor. Introduction à la psychanalyse. Paris: Petite Bibliothèque Payot (1915–1917). 337–55.

[3] Golse B. Le Modèle polyfactoriel en psychopathologie. In: Lavoisier, editor. *Traité européen de psychiatrie et de psychopathologie de l’enfant et de l’adolescent* (sous l’égide de l’AEPEA et sous la direction de P. FERRARI et O. BONNOT). Paris: Lavoisier, Médecine Sciences Publications (2013). 279–82.

[4] Bion W.R. (1963). Éléments de psychanalyse, P.U.F., Coll. Bibliothèque de Psychanalyse, Paris (1979) (lère éd).

[5] Keats J. Lettre à ses frères, 21 décembre 1817. In: Lavoisier, editor. *Keats and negative capability* (Keats et la capacité négative). Londres: Bloomsbury, coll. continuum literary studies (2011)

[6] Cooper RS. Gene-environment interactions and the etiology of common complex disease. Ann Intern Med. (2003) 139:437–40. doi: 10.7326/0003-4819-139-5_Part_2-200309021-00011 12965972

[7] Ramos RG Olden K. Gene-environment interactions in the development of complex disease phenotypes. Int J Environ Res Public Health. (2008) 5:4–11. doi: 10.3390/ijerph5010004 18441400 PMC3684407

[8] Golse B. *Le bébé et ses possibles*, Éditions Erès, Coll. « Thema/Psy ». Toulouse: (2019).

[9] Bernard CL. Introduction à l'étude de la médecine expérimentale (1865). Paris: Éditions Garnier-Flammarion (1865). 1966 p.

[10] Gorwood P Wohl M Purper D. Génétique des pathologies psychiatriques de l’enfant et de l’adolescent, EMC. Psychiatrie. (2004) 1:4–14. doi: 10.1016/j.emcps.2003.08.001

[11] Kanner L. Autistic disturbances of affective contact. Nervous Child. (1942) 3:217–30. 4880460

[12] Medjkane F Apter G. Épigénétique et autisme. Entre inné et acquis: un espace de convergence. Inf Psychiatr. (2014) 90:753–9.

[13] Bourgeron T Leboyer M Delorme R. Autisme, la piste génétique se confirme. Enfance. (2009) 1:93–8. doi: 10.3917/enf1.091.0093 19718887

[14] Jullien A. Autisme, crises convulsives, sclérose tubéreuse de Bourneville–Démarche diagnostique chez le jeune enfant et sa famille. Dev Dent. (2002) 14:121. doi: 10.3917/dev.022.0121

[15] Boissel L Le Borgne G Fuentealba Baldini L Gosme C Leitgel-Gille M Desguerre I. Attachment insecurity in infantile spasms: maternal anxiety and sadness and infant’s temperament outweigh disease severity. Epilepsy Behav. (2020) 113:107401. doi: 10.1016/j.yebeh.2020.107401, PMID: 33160148

[16] Ouss L Le Normand M-T Bailly K Leitgel-Gille M Gosme C Simas R. Developmental trajectories of hand movements in typical infants and those at risk of developmental disorders: an observational study of kinematics during the first year of life. Front Psychol. (2018) 9:83:83. doi: 10.3389/fpsyg.2018.00083, PMID: 29515472 PMC5826068

[17] Saitovitch A. Rechtman E. Vinçon-Leite A. Brunelle F. Zilbovicius M. Et Boddaert N., Imagerie dans l’autisme infantile, Encyclopédie Médico-Chirurgicale (EMC). Psychiatrie (2019), 16:11.

[18] Mottron L. L’autisme, une autre intelligence. Bruxelles: Mardaga, coll. Pratiques psychologiques (1990).

[19] Hochmann J. L’autisme infantile: déficit ou défense ? In: Parquet C l Golse BB, editors. Soigner, éduquer l’enfant autiste ? Paris: Masson, Coll. Médecine et Psychothérapie (1990). 33–55.

[20] Beversdorf D.. Communication à l’Ohio State University, le 21 (2001)

[21] Gallois TH Wendland J. Effets du stress prénatal sur le développement psychoaffectif: une revue de la question. Dev Dent. (2012) 24:245–62. doi: 10.3917/dev.123.0245

[22] Chamak B. Autism and social moverments: French paretns’associations and international autistic individuals’organisations. Sociol Health Illness. (2008) 30:76–96. doi: 10.1111/j.1467-9566.2007.01053.x, PMID: 18254834

[23] Chamak B. Autisme, handicap et mouvements sociaux. ALTER, Eur J Disab Res. (2010) 4:103–15. doi: 10.1016/j.alter.2010.02.001

[24] Spitz R. (1946). De la naissance à la parole–La première année de la vie. P.U.F., Coll. Bibliothèque de Psychanalyse: Paris (1979) (6).

[25] Kumsta R Kreppner J Kennedy M Knights N Rutter M Sonuga-Barke E. Psychological consequences of early global deprivation: an overview of findings from the English & Romanian Adoptees study. Eur Psychol. (2015) 20:138–51. doi: 10.1027/1016-9040/a000227

[26] Rutter M Andersen-Wood L Beckett C Bredenkamp D Castle J Groothues C. Quasi-autistic patterns following severe early global privation. J Child Psychol Psychiatry Allied Discip. (1999) 40:537–49. doi: 10.1111/1469-7610.00472, PMID: 10357161

[27] Rutter M Kreppner J Croft C Murin M Colvert E Beckett C. Early adolescent outcomes of institutionally deprived and non-deprived adoptees. III. Quasi-autism. J Child Psychol Psychiatry. (2007) 48:1200–7. doi: 10.1111/j.1469-7610.2007.01792.x, PMID: 18093025

[28] Tustin F. Les états autistiques chez l’enfant. Paris: Le Seuil (1986).

[29] Chamak B. L’autisme à Marseille Nord: inégalités territoriales, précarité et services publics. Neuropsychiatr Enfance Adolesc. (2019) 67:304–10. doi: 10.1016/j.neurenf.2019.03.004

[30] Aristote. (384–322 av. J.-C), Éthique à Nicomaque, Lgf, Livre de poche, Coll. Classiques Philo, Paris, (1992).

[31] Golse B Putois O Vanier A. (sous la direction de), Épistémologie et méthodologie en psychanalyse et en psychiatrie – Pour un vrai débat avec les neurosciences, Éditions Érès, Coll. Le Carnet-Psy. Toulouse: (2017).

